# Chemical constituents and coagulation activity of *Agastache rugosa*

**DOI:** 10.1186/s12906-017-1592-8

**Published:** 2017-02-06

**Authors:** Pengran Cao, Pingyao Xie, Xuebiao Wang, Jinmei Wang, Jinfeng Wei, Wen-yi Kang

**Affiliations:** 1 Institute of Chinese Materia Medica, Henan University, Kaifeng, 475004 China; 2 Kaifeng Key Laboratory of Functional Components in Health Food, Kaifeng, 475004 China

**Keywords:** *Agastache rugosa*, Chemical composition, Coagulant activity, Acacetin, Tilianin

## Abstract

**Background:**

In the Chinese traditional medicine, plant of *Agastache rugosa* (Fisch. & C.A. Mey.) Kuntze (*A. rugosa*) has been used to treat nausea, vomiting and dispel damp. However, currently, few reports about the chemical constituents, especially the non-volatile components of *A. rugosa* are available.

**Methods:**

Through separation with various column chromatographies to elucidate the chemical constituents of *A. rugosa*, the biological activities of the major constituents were investigated. The extracts and main constituents of *A. rugosa* were evaluated for their anticoagulant effects by assaying the activated partial thromboplastin time (APTT), prothrombin time (PT), thrombin time (TT) and fibrinogen (FIB) in vitro.

**Results:**

Seven known compounds (namely compounds **1**–**7**) were isolated from the aerial parts of *A. rugosa*. They were identified as methyl hexadecanoate (**1**), *β*-sitosterol (**2**), acacetin (**3**), ursolic acid (**4**), apigenin (**5**), protocatechuic acid (**6**) and tilianin (**7**), respectively. Compounds **1** and **6** were isolated from the genus *Agastache* for the first time, and compound **4** was obtained from the plants for the first time. The results showed that the extract of *A. rugosa* had a significant procoagulant activity by shortening the time of PT (*P* < 0.001) and increasing FIB content (*P* < 0.001), as compared with Vitamin K_1_. While its major constituents acacetin and tilianin exhibited significant anticoagulant activities by prolonging the times of PT, APTT, TT and reducing FIB content (*P* < 0.001), as compared with blank control group.

**Conclusions:**

The total extract of *A. rugosa* possessed significant procoagulant activity, while its main components, acacetin and tilianin possessed significant anticoagulant activities. Further investigation should be pursued to find out the bioactivity components responsible for the procoagulant action of the plant.

## Background

*Agastache rugosa* (*A. rugosa*), a medicinal plant belonging to the family Lamiaceae, is native to Korea, Japan, and China. *A. rugosa* shows the taste and thermal properties as pungent (acrid) is slightly warm and can the channel affiliations to enter spleen, stomach and lung. *A. rugosa* has been used in folk medicine to treat a variety of diseases, including cholera, vomiting, miasma and other intestinal disorders [1–4]. According to Traditional Chinese Medicine theory, *A. rugosa* is classified as an aromatic and damp dissolving herb. Thus it has been widely used as an effective herbal drug to cure the diseases of human pathogenic summer-heat and dampness virulence in clinical in China.

According to the records, the use of *A. rugosa* as a medicinal plant can be traced back to the Eastern Han dynasty (25 AD - 220 AD) in China. Pharmacological investigation showed that *A. rugosa* possessed antibacterial [5], anti-HIV integration activities [6], antioxidant [7], cardiovascular [8, 9], cytoprotective activity and other activities [10, 11]. Chemical studies showed that the chemical compositions of *A. rugosa* mainly contained essential oils, terpenoids, flavonoids and other constituents [11–13]. *A. rugosa* was collected in “Chinese Pharmacopoeia” as one of genuine medicinal materials before 1977, while “Chinese Pharmacopoeia” has only loaded *Pogostemon cablin* (Blanco) Benth since 1985. Because of this, *A. rugosa* was out of the scope of genuine medicinal materials, and thus the research interest in utilization of *A. rugosa* was reduced. Therefore, very few the studies on its chemical constituents and bioactivities have been available, in the recent years. In view of its abundant wild resource and diverse biological activities, it is necessary to study on its chemical constituents and to elucidate its chemical constituents and biological activities.

Thrombotic disease is a serious threat to human life and health and its incidence is ranked the highest one among various diseases. The incidence of thrombotic has still been displaying an increasing tendency. In the recent years, the studies on thrombotic disease are becoming one of the key points and hot spots in modern medicine. Drug therapy has played an important role in the prevention and treatment this disease. Currently, on the medicinal market, there are only three main types of anti-thrombotic drugs, including anticoagulant drugs, anti-platelet drugs and thrombolytic drugs. However, these drugs generally have greater adverse reactions and their prices are quiet high. Therefore, developing a safe, effective and low-cost anti thrombotic drug is an urgent need for clinical drug therapy for this disease.

Thrombosis is the proximate cause of some vascular disorders [14]. It has been shown that high-fat hyperlipidemia, obesity, hypertension, atherosclerosis, coronary heart disease, myocardial infarction, cerebral thrombosis and a series of cardiovascular and cerebrovascular diseases are associated with abnormal lipid metabolism. The main inducement include high levels of low-density lipoprotein-cholesterol (LDL-C), low levels of high density lipoprotein-cholesterol (HDL-C), the increase of free radicals and lipid peroxidation [15]. Recently, there are increasing interests in the biomedical field to isolate thrombolytic agents and anti-thrombotic compounds from food and natural sources, which are presumed to be safer and more effective. Since *A. rugosa* exhibits a cardiovascular protective effect, we hypothesized that *A. rugosa* may have anti-coagulation effect. A literature survey implicated that there have been no reports on the anti-coagulation activity of *A. rugosa.* Thus this study aimed to investigate the chemical constituents and the coagulation activity of *A. rugosa*.

## Methods

### Chemicals and material

^1^H NMR and ^13^C NMR spectra of chemical components of *A. rugosa* were measured on a Bruker AscendTM 400 spectrometer (Bruker Corporation, Billerica, MA, USA). Rotating evaporation instrument EYELA was purchased from Tokyo Physical and Chemical Equipment Co., Ltd. (Japan). A CXTH LC-3000 preparative HPLC system was obtained from Beijing Chuangxintongheng Science and Technology Co., Ltd. (China) and used for the high-performance column chromatography (HPLC) and equipped with binary pumps, an UV/VIS detector, and a manual injection valve. Preparative medium pressure liquid chromatographic (MPLC) separation was performed using a Sepacore glass column c-690 that was filled with Silica gel (60 mesh, 500 g). Silica gel GF 254 Thin Layer Chromatography (TLC) and Silica gel (40–80 mesh, 200–300 mesh) were purchased from Yantai Huiyou Development co., Ltd (Shandong, China). Silica gel H was made by Qingdao Haiyang Chemical Co., Ltd (Shandong, China). Column chromatography was performed over Sephadex LH-20 (Pharmacia Kalamazoo, MI, USA). Centrifugation was performed on Shanghai Anting TGL-16gr centrifugal.

### Plant material

*A. rugosa* (Fisch. et Mey.) O. Ktze was originally collected in October 2013 from the Suzhou region of Jiangsu Province, China and identified by Professor Changqin Li. A voucher specimen (201310231) was deposited in the Institute of Traditional Chinese Medicine, Henan University (Kaifeng, Henan, China).

### Extraction and isolation

The air-dried aerial parts of *A. rugosa* (2.8 Kg) were extracted with 70% ethanol at 50 °C (3 × 15 L, 6 h) to yield the crude extract (Ar.TE 178 g) after filtration and solvent evaporation using a rotary evaporator. The extract (178 g) was dissolved in H_2_O (500 mL), and extracted by petroleum, ethyl acetate and *n*-butanol in turn, and the recovered solvents were decompressed to obtain petroleum ether site (Ar.Pe, 11.8 g), ethyl acetate part (Ar.Ea, 19 g) and *n*-Butanol site (Ar.Bu, 25.2 g), respectively.

Ar.Ea was separated by medium pressure liquid chromatography (MPLC) that was filled with silica gel H, eluted with a stepwise-gradient, in sequence, of petroleum ether-ethyl acetate (100:1–1:1, v/v), chloroform-methanol (50:1–2:1, v/v) to afford 8 fractions (F_1_–F_8_) based on TLC analyses. F_2_ (1.54 g) was subjected to decompressed chromatographic column of silica gel H with a gradient of petroleum ether-CHCl_3_ (1:0–0:1, v/v) to afford 4 fractions (F_2–1_-F_2–4_). The separation of F_2–4_ using Sephadex LH-20 (petroleum ether/CHCl_3_/MeOH, 9:9:2, v/v/v) successively afforded compound **1** (25 mg) and F_2–4–1_. F_2–4–1_ was firstly subjected to ordinary pressure chromatographic columns of silica gel H with petroleum ether-CHCl_3_ (1:10–0:1, v/v) and then through re-crystal to obtain compound **2** (28.7 mg). F_4_ (1.72 g) was subjected to decompressed chromatographic column of silica gel H with petroleum ether-ethyl acetate (40:1–0:1, v/v) and then separated using Sephadex LH-20 (CHCl_3_/MeOH, 1:1, v/v) to obtain compound **3** (15.8 mg). F_5_ was subjected to decompressed chromatographic column of silica gel H with petroleum ether-ethyl acetate (10:1–0:1, v:v) to obtain F_a_ and F_b_. F_b_ was subjected to decompressed chromatographic column of silica gel H with CHCl_3_-acetone (1:0–1:1, v/v) and Sephadex LH-20 (CHCl_3_/MeOH, 1:1, v/v) to obtain compounds **3** (16.3 mg) and compounds **4** (33.4 mg). Compounds **3** (63.8 mg) and **4** (145.7 mg) were enriched in the same way from F_6_ and F_7_. F_7_ was subjected to decompressed chromatographic column of silica gel H with CHCl_3_/acetone (50:1–0:1, v:v) to obtain F_7–1_ and F_7–2_, and then F_7–1_ was separated using Sephadex LH-20 (CHCl_3_/MeOH, 1:1, v/v) to obtain compound **5** (3 mg). F_7–1_ was subjected to decompressed chromatographic column of silica gel H with CHCl_3_-MeOH (1:0–1:1, v/v) and Sephadex LH-20 (CHCl_3_/MeOH, 1:1, v/v) to obtain compound **6** (21.4 mg).

Ar.Bu was separated by MPLC that was filled with silica gel H, eluted with CHCl_2_-MeOH (1:0–1:1, v/v) to afford 7 parts (P_1_–P_7_) based on TLC analyses. P_1_ was separated with Sephadex LH-20 (CHCl_2_/MeOH, 1:1, v/v) to obtain compound **3** (35.8 mg). The solubility of P_3_ was not good. P_3_ was washed with petroleum ether, CHCl_2_, ethyl acetate, acetone and methanol and mixed solvent of various proportions, to afford un-dissolved substance compound **7** (235.3 mg).

### The Activated Partial thromboplastin time (APTT), Prothrombin Time (PT), Thrombin Time (TT) and Fibrinogen (FIB) Assays in Vitro

Blood samples were drawn from Rabbit’s Auricular Vein (NO: 2015–035). Before collection, the sodium citrate (38 mg/mL, 400 μL) was placed in a 4 mL centrifuge tube to prevent blood clotting. Plasma was then separated from the blood by centrifugation at 3000 rpm 5 °C for 15 min. APTT and PT were determined according to the method described previously [16]. In brief, plasma (100 μL) was mixed with 20 μL of samples, APTT assay reagent (100 μL) was added and incubated for 5 min at 37 °C, and then 25 mM CaCl_2_ (100 μL) was added. Clotting times were recorded. For PT assays, plasma (100 μL) was mixed with 20 μL of samples and incubated at 37 °C for 3 min. PT assay reagent (200 μL), which was hatched at 37 °C for 3 min, was then added and clotting time was recorded. TT and FIB were determined according to the manufacturer’s recommendations (Shanghai Sun Biotech Co., Ltd, China).

For all the tests mentioned above, blank solvent (dimethyl sulphoxide: Tween 80: normal saline = 2:1:17) was used as negative control group, while the drugs of breviscapine (13.3 mg/mL) and Vitamin K_1_ (5 mg/mL) used in clinical were used as positive control groups. All the samples were dissolved in blank solvent. The concentrations of compounds were 5 mg/mL and all the extract samples were 15 mg/mL. PT, APTT, TT and FIB tests were conducted by Semi-Automated Coagulation Analyzer (CPC Diagnostics Pvt. Ltd, India).

### Statistical analysis

The results were expressed as the arithmetic mean plus or minus standard deviation. Numerical statistics were performed using SPSS19.0 software with single factor analysis of variance (ANOVA One-Way) to determine the significant difference. The difference between groups with *P* < 0.05 and *P* < 0.001 were regarded as significant and highly significant, respectively. Results were shown in Table 1.

**Table 1:** The effects of *A. rugosa* extracts﻿, acacetin and tilianin on APTT, PT, TT, and FIB in vitro (‾*x* ± *s*)

Groups	APTT (S)	PT (S)	TT (S)	FIB (g/L)
Blank	19.5000 ± 0.216	11.5750 ± 0.275	14.2750 ± 0.13	3.7250 ± 0.132
Breviscapine	22.5000 ± 0.216^***^	14.0000 ± 0.163^***^	20.7750 ± 0.15^***^	6.1633 ± 0.035^***^
Vitamin K_1_	16.5500 ± 0.129^***###^	10.9333 ± 0.115^***###^	13.5000 ± 0.26^***###^	4.6000 ± 0.145^***###^
Ar.TE	19.3750 ± 0.320^###&&&^	10.5000 ± 0.258^***###&^	14.5250 ± 0.19^##&&&^	6.7150 ± 0.153^***###&&&^
Ar.Pe	16.0500 ± 0.238^***###&&^	10.2500 ± 0.191^***###&&&^	18.6475 ± 0.20^***###&&&^	3.8350 ± 0.066^###&&&^
Ar.Ea	16.625 ± 0.222^***###^	11.0500 ± 0.238^**###^	17.2450 ± 0.19^***###&&&^	3.7175 ± 0.111^###&&&^
Ar.Bu	16.0967 ± 0.295^***###&^	10.8750 ± 0.275^***###^	15.2750 ± 0.25^***&&&^	3.9100 ± 0.071^**###&&&^
Acacetin	20.7225 ± 0.121^***###&&&^	12.5500 ± 0.311^***###&&&^	20.2750 ± 0.26^***##&&&^	2.8125 ± 0.142^***###&&&^
Tilianin	20.7500 ± 0.191^***###&&&^	12.2000 ± 0.082^***###&&&^	16.7475 ± 0.19^***###&&&^	3.1425 ± 0.111^***###&&&^

*Note*: results were expressed as mean ± SD, *n* = 4. Compared with blank: ^***^ *P* < 0.001; 0.001 < ^**^ *P* < 0.01; Compared with breviscapine: ^###^ *P* < 0.001; 0.001 < ^##^ *P* < 0.01. Compared with Vitamin K_1_: &&&*P* < 0.001, 0.001 < &&*P* < 0.01, &*P* < 0.05

## Results

### Chemical constituents in *A. rugosa*

Compound **1**: was colorless oily liquid with melting point (mp) at 28–30 °C. The molecular formula was determined to be C_17_H_34_O_2_. EI-MS *m/z*: 270[M]^+^. ^1^H-NMR (CDCl_3_, 400 MHz) *δ*: 0.87 (3H, t, *J* = 8Hz, 4Hz, H-16), 1.25 (24H, s, H-4–15), 1.61 (2H, m, H-3), 2.29 (2H, t, *J* = 8 Hz, 8Hz, H-2), 3.66 (2H, s, H-1′). ^13^C-NMR (CDCl_3_, 100 MHz) *δ*: 174.2 (C-1), 51.35 (C-1′), 34.11 (C-2′), 24.96 (C-3), 29.15 (C-4), 29.24 (C-5), 29.34 (C-6), 29.67 (C-7, C-11), 29.58 (C-12), 29.44 (C-13), 31.91 (C-14), 22.67 (C-15), 14.06 (C-16). The above spectral data were basically consistent with those reported previously [17] and thus, compound **1** was identified as methyl hexadecanoate.

Compound **2**: this compound was a white powder with mp at 136–137 °C. The molecular formula was determined to be C_29_H_50_O. EI-MS *m/z*: 414[M]^+^. When compound **2** was compared with reference substance of *β*-sitosterol, no difference was seen between them in term of the TLC detection. Its *R*_*f*_ values were the same in three different developing agents, and the coloration were the same. The samples were mixed with reference substance of *β*-sitosterol, and the melting point of the mixture was not decreased. Thus compound **2** was identified as *β*-sitosterol.

Compound **3**: this compound is a yellow, needle-shaped crystal with mp at 261–262 °C. The molecular formula was determined to be C_16_H_12_O_5_. EI-MS *m/z*: 283 [M-H]^−^. ^1^H-NMR (DMSO-*d*_*6*_, 400 MHz) *δ*: 12.88 (1H, s, 5-OH), 8.01 (2H, d, *J* = 8.0 Hz, H-2′, 6′), 7.11 (2H, d, *J* = 8.0Hz, H-3′, 5′), 6.82 (1H, s, H-3), 6.50 (1H, s, H-8), 6.20 (1H, s, H-6), 3.84 (3H, s, −OCH_3_). ^13^C-NMR (DMSO-*d*_*6*_, 100 MHz) *δ*: 122.98 (C-1), 163.61 (C-2), 103.67 (C-3), 181.97 (C-4), 157.57 (C-5), 94.29 (C-6), 164.45 (C-7), 99.14 (C-8), 162.54 (C-9), 103.94 (C-10), 161.61 (C-4′), 128.52 (C-2′, 6′), 114.82 (C-3′, 5′), 55.75 (4′-OCH_3_). The above data were basically consistent with those reported in the reference [18]. Thus, compound **3** was identified as acacetin.

Compound **4**: this compound was a white crystalline with mp at 240–245 °C. The molecular formula was determined to be C_30_H_48_O_3_. EI-MS *m/z*: 456[M]^+^. ^1^H-NMR (C_5_D_5_N, 400 MHz) *δ*: 5.50 (1H, s, H-12), 3.46 (1H, dd, *J* = 4.0, 8.0Hz, H-3), 2.33 (1H, q, H-18), 1.25 (3H, s, H-27), 1.24 (3H, s, H-26), 1.06 (3H, s, H-23), 0.95 (3H, d, *J* = 8.0Hz, H-29), 0.90 (3H, s, H-24), 1.02 (3H, d, *J* = 8.0Hz, H-30), 1.03 (3H, s, H-25). ^13^C-NMR (C_5_D_5_N, 100 MHz) *δ*: 37.17 (C-1), 27.85 (C-2), 77.88 (C-3), 38.84 (C-4), 55.57 (C-5), 18.52 (C-6), 33.32 (C-7), 39.73 (C-8), 47.78 (C-9), 39.23 (C-10), 23.64 (C-11), 125.38 (C-12), 138.99 (C-13), 42.25 (C-14), 28.53 (C-15), 24.65 (C-16), 47.78 (C-17), 53.30 (C-18), 39.14 (C-19), 39.09 (C-20), 30.81 (C-21), 37.03 (C-22), 28.43 (C-23), 15.40 (C-24), 16.27 (C-25), 17.23(C-26), 23.36 (C-27), 179.55 (C-28), 17.18 (C-29), 21.11 (C-30). The above data were basically consistent with those reported in the reference [19]. Thus, compound **4** was identified as ursolic acid.

Compound **5**: this compound was a yellow, needle-shaped crystal with mp at 275–276 °C. The molecular formula was determined as C_15_H_10_O_5_. EI-MS *m/z*: 270 [M]^−^。^1^H-NMR (DMSO-*d*_*6*_, 400 MHz) *δ*: 12.95 (1H, s, 5-OH), 10.81 (1H, s, 7-OH), 10.33 (1H, s, 4′-OH), 7.91 (2H, d, *J* = 8.0 Hz, H-2′, 6′), 6.92 (2H, d, *J* = 4.0 Hz, H-3′, 5′), 6.77 (1H, s, H-3), 6.48 (1H, s, H-8), 6.19 (1H, s, H-6). ^13^C-NMR (DMSO-*d*_*6*_, 100 MHz) *δ*: 164.10 (C-2), 102.83(C-3), 181.72 (C-4), 161.43 (C-5), 98.82 (C-6), 163.74 (C-7), 93.94 (C-8), 157.29 (C-9), 103.68 (C-10), 121.17 (C-1′), 128.44 (C-2′, 6′), 115.94 (C-3′, 5′), 161.14 (C-4′). The above data were basically consistent with those reported in reference [18]. Thus the compound **5** was identified as apigenin.

Compound **6**: this compound was a yellow-brown, needle-shaped crystal with mp at 199–200 °C. The molecular formula was determined as (HO)_2_C_6_H_3_COOH. EI-MS *m/z*: 153[M]^−^. ^1^H-NMR (DMSO-*d*_*6*_, 400 MHz) *δ*: 7.33 (1H, s, H-2), 7.29 (1H, d, *J* = 8.0 Hz, 6-H), 6.79 (1H, d, *J* = 8.0Hz, 5-H). ^13^C-NMR (DMSO-*d*_*6*_, 100 MHz) *δ*: 121.87 (C-1), 116.74 (C-2), 145.03 (C-3), 150.15 (C-4), 115.31 (C-5), 122.03 (C-6), 167.43 (C-7). The above data were basically consistent with those reported in the reference [20]. Thus, the compound **6** was identified as protocatechuic acid.

**Fig. 1:**
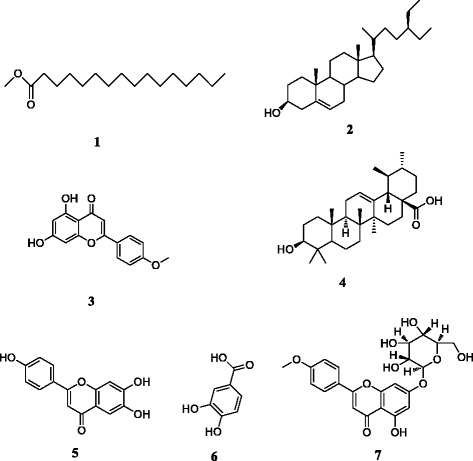
Structures of compound **1** ~ **7**

Compound **7**: this compound was a light yellow powder with mp at 256–257 °C. The molecular formula was determined to be C_22_H_22_O_10_. EI-MS *m/z*: 446[M]^+^. ^1^H-NMR (DMSO-*d*_*6*_, 400 MHz) *δ*: 12.91 (1H, s, 5-OH), 8.07 (2H, d, *J* = 8.0 Hz, H-2′, 6′), 7.14 (2H, d, *J* = 8.0 Hz, H-3′, 5′), 6.95 (1H, s, H-3), 6.86 (1H, s, H-8), 6.46 (1H, s, H-6), 5.06 (1H, d, *J* = 8.0Hz, Glc-H-1′′), 3.71 (1H, m, Glc-H-2′′), 3.18 (1H, m, Glc-H-3′′), 3.45 (1H, m, Glc-H-4′′), 3.47 (1H, m, Glc-H-6′′b). ^13^C-NMR (DMSO-*d*_*6*_, 100 MHz) *δ*: 163.00 (C-2), 103.78 (C-3), 181.98 (C-4), 156.93 (C-5), 99.56 (C-6), 163.80 (C-7), 94.90 (C-8), 162.44 (C-9), 105.36 (C-10), 122.66 (C-1′), 128.39 (C-2′, 6′), 161.07 (C-4′), 114.59 (C-3′, 5′), 55.54 (4′-OCH_3_), 99.94 (C-2′′), 73.08 (C-2′′), 77.15 (C-3′′), 69.57 (C-4′′), 76.41 (C-5′′), 60.60 (C-6′′). The above data were basically consistent with those reported in the reference [21]. Thus, compound **7** was identified as tilianin. All the sructures of compound 1 ~ 7 were shown in Fig. 1.

### Coagulation time test in vitro

**Fig. 2:**
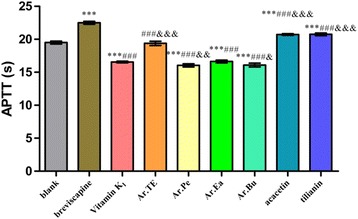
The effects of *A. rugosa* extract, acacetin and tilianin on APTT in vitro

In Fig. 2, compared with the blank group, both acacetin and tilianin could significantly prolong APTT (*P* < 0.001) while Ar.Pe, Ar.Ea, and Ar.Bu had highly significant effects on promoting blood coagulation (*P* < 0.001). Compared with the breviscapine, the effects of acacetin and tilianin were not better than that of the positive control (*P* < 0.001). Compared with the Vitamin K_1_, Ar.Pe and Ar.Bu had significant effects on promoting coagulation (0.01 < *P* < 0.001 and *P* < 0.05, respectively).

**Fig. 3:**
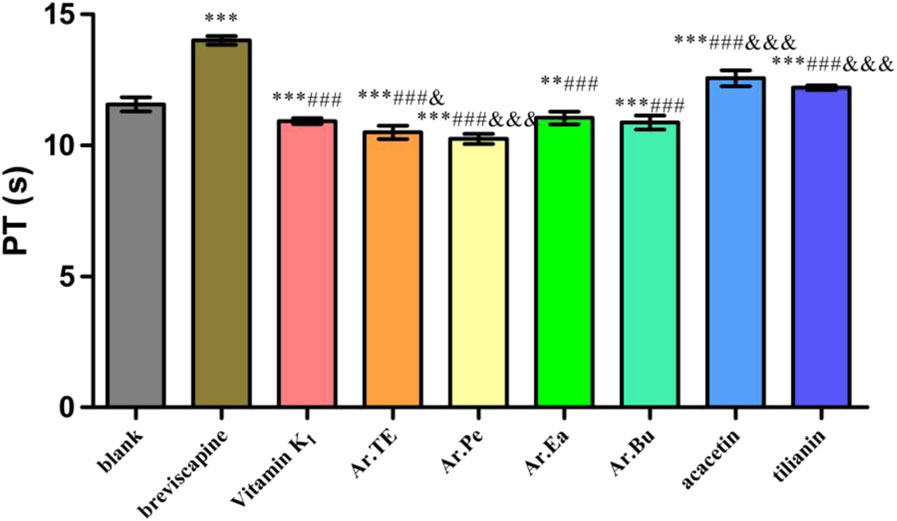
The effects of *A. rugosa* extract, acacetin and tilianin on PT in vitro

Results shown in the Fig. 3 indicated that compared with the blank group, acacetin and tilianin could significantly prolong PT (*P* < 0.001) while Ar.TE, Ar.Pe, Ar.Ea, Ar.Bu had highly significant effects on promoting blood coagulation (*P* < 0.001 and 0.001 < *P* < 0.01, respectively). Compared with the breviscapine, the effects of acacetin and tilianin were not better than the positive control (*P* < 0.001). Compared with the Vitamin K_1_, Ar.TE and Ar.Pe had significant effects on promoting coagulation (*P* < 0.05 and *P* < 0.001, respectively).

**Fig. 4:**
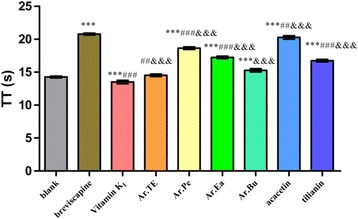
The effects of *A. rugosa* extract, acacetin and tilianin on TT in vitro

As shown in Fig. 4, compared with the blank group, Ar.Pe, Ar.Ea, Ar.Bu, acacetin and tilianin could significantly prolong TT (*P* < 0.001). Compared with the breviscapine, the anticoagulant activity of the positive control was the best one (*P* < 0.001).

**Fig. 5:**
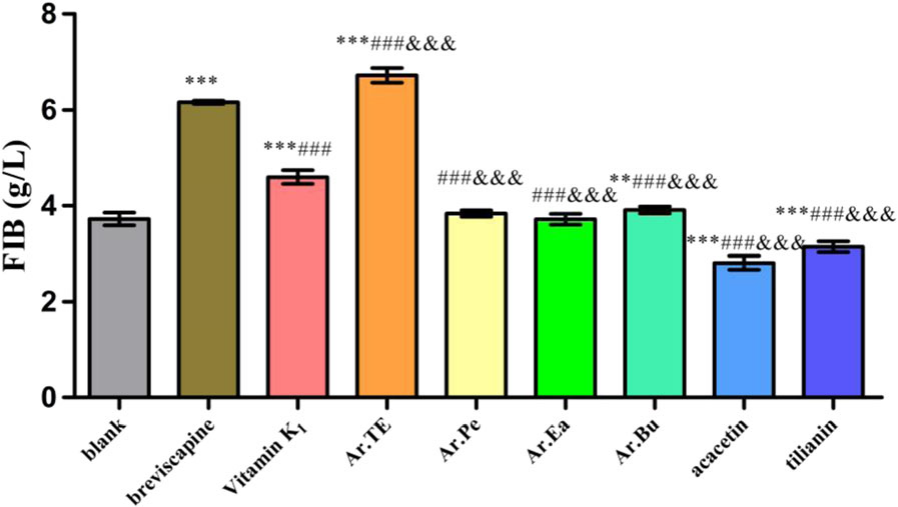
The effects of *A. rugosa* extract, acacetin and tilianin on FIB in vitro

Results shown in the Fig. 5 indicated that compared with the blank group, acacetin and tilianin could significantly decrease the FIB content (*P* < 0.001). Compared with the Vitamin K_1_, Ar.TE could significantly increase FIB content (*P* < 0.001).

## Discussion

Through the chemical analysis, we found that ursolic acid, acacetin and tilianin were the main chemical components in *A. rugosa*. These compounds have been known to have a wide range of biological activities and play a role in triggering cardiovascular activities. For instance, Li et al. [22] reported that ursolic acid could promote the neuroprotection by activating nuclear factor-erythroid 2-related factor-2 pathway after cerebral ischemia in mice. Yang et al. [23] reported that ursolic acid could reestablish the intracellular redox state. Li et al. [24] demonstrated that the natural compound acacetin was an atrium-selective agent that prolonged the atrial effective refractory period without prolonging the corrected Q wave - T wave (QT) interval and effectively prevented atrial fibrillation (AF) in anesthetized dogs after intraduodenal administration. Tilianin mediated relaxation mainly by an endothelium-dependent manner, probably due to NO release, and also through an endothelium-independent pathway by opening K^+^ channels, both could cause the antihypertensive effect [25].

There have been no reports in the literature about the anticoagulant activity of *A. rugosa*. In this study, we found that the main chemical components, including ursolic acid, acacetin and tilianin of *A. rugosa* had anticoagulant-related activity. Anticoagulant activities of Ar.TE, Ar.Pe, Ar.Ea, Ar.Bu, acacetin and tilianin were investigated in the experiment in vitro.

The results obtained in this study are quite interesting. Firstly, Ar.TE had an activity of promoting coagulation as shown by PT and FIB while Ar.Pe, Ar.Ea and Ar.Bu had procoagulant activities as shown by APTT and PT. Acacetin, ursolic acid and tilianin all displayed significant anticoagulant activities as shown by PT, APTT, TT and FIB. The activities of Ar.Pe, Ar.Ea and Ar.Bu were different because their main components were different. For instance, acacetin and ursolic acid were the main components in the Ar.Ea while tilianin was one of the main components in the Ar.Bu. The difference in main components might be due to following reasons: the compounds having a great effect might not be isolated or the remaining compounds could have a great effect on promoting coagulant, though their contents might be very low. The chemical components of plant medicines are complex, and the pharmacological effects can be the interactions of multi-components, multi targets together, either synergistically or antagonistically. There might be no any compounds having great effects on promoting coagulation. This is the common function of several components.

As compared with the blank group, both acacetin and tilianin exhibited significant anticoagulant activities as demonstrated by PT, APTT, TT and FIB assays. Acacetin and tilianin are natural flavonoid compounds with abundant sources from plant origin. The techniques for isolation and separation of both compounds are simple and easy to operate. The acute toxicity experiment of acacetin showed that it was a safe clinical medication (LD_50_ = 933 mg/kg) [26]. The acute toxicity test of tilianin had not been done yet and there was no report about its toxicity. Therefore, both acacetin and tilianin may have a good pharmaceutical prospect. Being cheap, safe and effective, they are in line with the requirements of today’s market fort anti-thrombotic drugs. Of course, the mechanism underlying their anti-coagulation activity need to be further investigated.

*A. rugosa* has been commonly used to treat summer heat-dampness and stomach flu etc. Because *A. rugosa* is also an aromatic plant, the studies on *A. rugosa* have been mostly focused on the volatile components. The research on non volatile component of *A. rugosa* was mainly initiated in the 1990s [27–30], and until recently, there were have been few studies on the chemical composition and biological activity of *A. rugosa.* Thus, we investigated the chemical composition and coagulation activity of *A. rugosa* and found that Ar.TE had a significant procoagulant activity, compared with Vitamin K_1_. Acacetin and tilianin had significant anticoagulant activity, as compared with the blank group. Further investigation should be pursued to find the effect of *A. rugosa*, acacetin and tilianin in vivo. Since the effects of acacetin and tilianin are different from those of *A. rugosa*, it should be noted that other active components in the extract remained to be further identified and characterized.

## Conclusions

*A. rugosa* has been a traditional Chinese medicinal plant. The previous studies on *A. rugosa* were mainly focused on the volatile components and its traditional pharmacological activity. In the present study, we found for the first time that the *A. rugosa* had a significant coagulant activity. The total extract of *A. rugosa* possessed significant procoagulant activity while acacetin and tilianin identified as the main components of *A. rugosa* had a significant anticoagulant activity in vitro. As a novel, effective and promising drug for the treatment of various coagulation disorders, *A. rugosa* may be beneficial for the individual with high risks of with hemophilia and other bleeding disorders. Acacetin and tilianin were natural flavonoid compounds, whose plant origin was abundant and isolation and separation technique were simple and easy to operate. They might have a good pharmaceutical prospect. Being cheap, safe and effective, they can meet requirements of today’s market about antithrombotic drugs.
